# Implementation of the Blended Care Self-Management Program for Caregivers of People With Early-Stage Dementia (Partner in Balance): Process Evaluation of a Randomized Controlled Trial

**DOI:** 10.2196/jmir.7666

**Published:** 2017-12-19

**Authors:** Lizzy MM Boots, Marjolein E de Vugt, Claudia MJ Smeets, Gertrudis IJM Kempen, Frans RJ Verhey

**Affiliations:** ^1^ Department of Psychiatry and Neuropsychology Alzheimer Center Limburg School for Mental Health and Neurosciences, Maastricht University Medical Center+ Maastricht Netherlands; ^2^ Department of Health Services Research Care and Public Health Research Institute Maastricht University Maastricht Netherlands

**Keywords:** internet, caregivers, technology, therapeutics

## Abstract

**Background:**

Caring for a family member with dementia puts caregivers at risk of overburdening. Electronic health (eHealth) support for caregivers offers an opportunity for accessible tailored interventions. The blended care self-management program “Partner in Balance” (PiB) for early-stage dementia caregivers was executed in Dutch dementia care organizations. The program combines face-to-face coaching with tailored Web-based modules. Next to an evaluation of program effectiveness, an evaluation of sampling and intervention quality is essential for the generalizability and interpretation of results.

**Objective:**

The aim of this study was to describe the process evaluation from the perspective of both family caregivers (participants) and professionals delivering the intervention (coaches) to determine internal and external validity before the effect analysis and aid future implementation.

**Methods:**

Implementation, sampling, and intervention quality were evaluated with quantitative and qualitative data from logistical research data, coach questionnaires (n=13), and interviews with coaches (n=10) and participants (n=49). Goal attainment scaling was used to measure treatment-induced change. Analyses were performed with descriptive statistics and deductive content analysis.

**Results:**

The participation rate of eligible caregivers was 51.9% (80/154). Recruitment barriers were lack of computer and lack of need for support. Young age and employment were considered recruitment facilitators. All coaches attended training and supervision in blended care self-management. Deviations from the structured protocol were reported on intervention time, structure, and feedback. Coaches described an intensified relationship with the caregiver post intervention. Caregivers appreciated the tailored content and positive feedback. The blended structure increased their openness. The discussion forum was appreciated less. Overall, personal goals were attained after the program (T>50). Implementation barriers included lack of financing, time, and deviating target population.

**Conclusions:**

Participants and coaches were satisfied with the intervention, but adapting the content to specific subgroups, for example, younger caregivers, was recommended. Implementation of the program requires more awareness of the benefits of blended care self-management programs and training in tailored self-management skills.

**Trial Registration:**

Dutch Trial Register (NTR): NTR4748; http://www.trialregister.nl (Archived by WebCite at http://www.webcitation.org/6vSb2t9Mg)

## Introduction

### Informal Dementia Caregivers Under Pressure

Dementia is a syndrome that causes deterioration in cognitive functioning. It affects memory, thinking, orientation, comprehension, learning capacity, language, and judgment, and is often accompanied by deterioration of emotional control, social behavior, or motivation. The impairment is severe enough to interfere with daily life. Dementia is caused by various diseases and injuries that primarily or secondarily affect the brain, such as Alzheimer’s disease or stroke [1]. Most people with dementia live at home and are cared for by one or more family members, often referred to as the informal caregivers [2]. Caring for a family member with dementia puts one at risk for depression, anxiety, and other health problems [3]. The benefits of electronic health (eHealth) support for caregivers are increasingly recognized in dementia care practice because it offers an opportunity for accessible tailored interventions. Furthermore, eHealth interventions may fill the expected gap in supply and demand caused by demographic aging and the decreasing working population [2,4].

### Caregiver Support

Previous research has found that specifically multicomponent, tailored eHealth interventions are promising for increasing caregiver confidence and self-efficacy and decreasing caregiver stress and depression [5,6]. Furthermore, blending online and face-to-face support may increase caregiver-therapist relations and adherence [7]. Next to intervention delivery, intervention timing might be crucial for efficacy. Early support may help people with dementia and their caregivers adapt to the changes of early dementia and feel more competent in their care skills. However, the current contrast between highly advanced timely diagnostic tools and the lack of available support to match this early diagnosis is troubling [8]. Early interventions can offer support in coping with an insecure future and preparing caregivers for the possibility of further decline and dependency [9] and have been proved to be effective in reducing strain and delaying institutionalization of the person with dementia [10,11]. However, some caregivers may feel stigmatized and refuse help if the information does not fit their personal situation and stage of the disease [12]. Existing programs are mostly aimed at problematic behaviors that occur in the advanced stages of dementia [13-17], or are not specifically adapted to the needs of caregivers during the early stages of dementia [18]. Early-stage interventions may prove to be beneficial if they focus on adaptation to the caregiver role and actively involve caregivers to tailor the intervention to their needs [9,19].

### Intervention Development, Evaluation, and Implementation

The Medical Research Council (MRC) framework suggests that intervention developers should put effort into the actual use of effective interventions by considering the implementation during the first phases of development and evaluation [20]. Following these recommendations, the blended care program “Partner in Balance” (PiB) for early-stage dementia caregivers was developed together with potential users. The program focuses on caregivers’ capacity to fulfill their potential and obligations and to help them manage their lives with some degree of independence and to engage in social activities together with the care recipient [21], fitting the new definition of health for chronic conditions [22]. To evaluate the program’s effectiveness, a randomized controlled trial (RCT) was performed in the context of daily care practice. Several caregiver eHealth support studies lack methodological rigor [6], and RCTs are considered proper designs for convincing evidence. However, before the effect analysis, evaluation of the sampling quality and intervention quality is essential for the interpretation and generalizability of results and further fine-tuning (or even annulling) of the effect analysis [23]. Furthermore, knowledge of implementation barriers and facilitators in an effect study can aid future adoption and implementation of a new intervention in care practice. Generalization and applicability of findings, for example, the public health significance of interventions (external validity), are particularly of interest for clinicians and policy makers. Implementation can be complicated in the complex and heterogeneous structure and culture of dementia care organizations [24]. Furthermore, the Consolidated Standards of Reporting Trial (CONSORT)-EHEALTH reporting standards for eHealth interventions place additional focus on external validity by assessing the following: (1) the context within participants benefitting from the intervention; (2) the delivery mode, features, and functionality of the intervention; (3) the use of prompts to interact with the intervention; and (4) any cointerventions that may occur. Additionally, a newly developed program can be effective but difficult to implement in care practice if health professionals or policy makers do not accept it. The implementation aspects (ie, costs and intervention description, including frequency, type, and duration of contacts) are important according to the CONSORT-EHEALTH standards. Therefore, process evaluations should focus on internal validity and implementation knowledge to increase credibility [23].

### Aim of This Study

Different frameworks for process evaluations have been proposed, but there is no consensus on elements that process evaluations should cover [23]. In this study, we describe first-order (sampling and intervention quality) and second-order (implementation knowledge) process data based on the model of Leontjevas et al [23] to assess the internal and external validity of the PiB trial and its implementation to inform effect analysis. Similar to the Reach Effectiveness Adoption Implementation Maintenance (RE-AIM) model, this model included the recommendations fitting the CONSORT-EHEALTH standards defining the internal and external validity.

## Methods

### Ethical Approval

The Medical Ethics Committee of the Maastricht University Medical Center+ (MUMC+) approved this study (#12-4-059). The process evaluation was conducted alongside the effectiveness study. Detailed information on the methods are presented in a previous study [25]. A short description is provided below.

### Study Design

A randomized waiting-list controlled trial was conducted in the southern part of the Netherlands. Family caregivers were recruited within memory clinics, mental health care organizations, and caregiver support services. The intervention was delivered and evaluated within these organizations. A waiting-list controlled design was chosen to increase acceptability and adherence to the research protocol in the control group and decrease attrition effects [20]. Data were collected pre- and postintervention and at 3-, 6-, and 12-month follow-up points.

### Intervention

Detailed information about the program components and development is presented in a previous study [21]. In short, the blended care self-management program PiB consists of the following: (1) a face-to-face intake session with a personal coach to familiarize participants with the program, set goals with the goal attainment scaling (GAS) method, and select preferred module themes [26]; (2) tailored online thematic modules, including psychoeducation, behavioral modeling, reflective assignments, change plans, and email feedback from the coach over 8 weeks; and (3) a face-to-face evaluation session with the coach evaluating previously set goals. Furthermore, the participants can interact with other participants via the discussion forum. The participants are free to set their own personal goals. The module themes are acceptance, balance in activities, communication with family members and environment, coping with stress, focusing on the positive, insecurities and rumination, self-understanding, the changing family member, and social relations and support. The participants choose 4 modules; 2 weeks were allocated for each module. However, the participants were allowed to complete the modules at their own pace in accordance with the self-management approach [27]. The personal page and modules remained accessible for participants after the intervention period. The control group consisted of an 8-week waiting list while receiving usual care (nonfrequent counseling). After the posttest assessment, they were given the opportunity to follow PiB.

### The Personal Coach

The personal coaches were trained, experienced professionals (psychologist or psychiatric nurses) from one of the participating organizations. They attended a 2-hour training session in self-management techniques, goal setting and online help, and regular supervision meetings. Their tasks were familiarizing participants with the online program, supporting them in module choice and goal setting, and giving feedback on the self-reflective assignments through the online messaging portal of the program, which was accessed via email.

### Process Evaluation

First-order (sampling and intervention quality) and second-order process data (implementation knowledge) were collected following a process evaluation framework based on earlier research [23,24].

#### First-Order Process Data: Sampling and Intervention Quality

The sampling quality was determined by a description of the procedures of recruitment, informed consent (IC), allocation, recruitment barriers and facilitators, and reach. Data were extracted from the research database and 2 focus group interviews with 5 coaches per group (Textbox 1).

**Topic list for individual interviews with participants and coaches.**

Individual interview participants (n=49)

- Clarity of content
- Websites’ ease of use
- Satisfaction with blended care (online and face-to-face)
- Satisfaction with personal coach and feedback
- Application and impact on daily life
- Advantages and disadvantages
- Recommendations

Focus group interview coaches (n=10)

- Feasibility: recruitment, instructions, time investment, and collaboration researchers
- Value for participating caregivers
- Barriers and facilitators of program implementation

**Table 1:** Demographic characteristics of interview participants at T0.

Demographic characteristics (N=49)		Value
Age in years, mean (SD)^a^		69.6 (9.2)
Spouse, mean (%)		48 (98)
Care intensity in years, mean (SD)		1.9 (1.8)
Female, n (%)		35 (71.4)
**Education, n (%)**		
	Primary education	8 (16.3)
	High school	17 (34.7)
	College	24 (49.0)
**Care recipients’ stage of dementia, n (%)**^b^		
	Preclinical	31 (63.3)
	Mild	16 (32.7)
	Moderate	2 (4.0)

^a^SD: standard deviation.

^b^As measured by the Global Deterioration Scale [28].

The information on intervention quality (relevance, feasibility, and performance according to protocol) was gathered from the perspective of both *coaches* and *participants*. Data collection from the perspective of coaches involved the registration of protocol deviations plus the amount and intensity of contact with caregivers on a structured registration form (Multimedia Appendix 1), an 8-item questionnaire rating the overall usability of PiB and its relevance for caregivers and coaches, with 4 multiple-choice items rated on a 5-point scale (1=completely disagree to 5=completely agree) (Multimedia Appendix 2) and 4 open-ended items on advantages, disadvantages, recommendations for other organizations or colleagues, and general appreciation of the program. Data from the perspective of the participants were collected postintervention with a semistructured interview (Textbox 1) with participants in both the intervention and waiting-list group.

Table 1 shows the demographic characteristics of the caregivers who participated in the interview.

The interviews were audiotaped with the participants’ permission. GAS was used to measure treatment-induced change and to compare the relative success of previously set personal goals [26]. GAS is a suitable measure to translate goals into achievement ratings. The scores range from −2 (much less than expected) to +2 (much better than expected), with a score of 0 meaning that the goal was attained. Furthermore, usage of the website (clickstream per intervention component) was tracked. Clickstream data are information trails that users leave behind while visiting the website. As participants clicked anywhere on the webpage, this action was captured in a log file. Clicks represent the number of times a page has been viewed and can be used to track which elements of the website were visited most often [29].

#### Second-Order Process Data: Implementation Knowledge

Knowledge on implementation of the program (components delivered and received, barriers and facilitators to implementation) was investigated with additional items in the questionnaire for the coaches described above (2 open-ended items on barriers and facilitators for program implementation; Multimedia Appendix 2) and the data obtained from the focus group interviews (Textbox 1).

### Analysis

Descriptive statistics (SPSS Statistics V22.0, IBM USA, NY, 2013) were used for the quantitative data analysis. The interviews were audiotaped and transcribed verbatim. The deductive content analysis was used for the qualitative data analysis (open-ended questions and transcripts) by authors LB and CS using the qualitative analysis software ATLAS.ti (V1.0.4, GmbH Germany: Berlin, 2014). The first author developed an unconstrained analysis matrix based on the interview themes. Both authors (LB and CS) coded the data for correspondence with or exemplification of the identified categories. Data not fitting the unconstrained matrix served as the basis for additional categories created following the principles of inductive content analysis; conceptual labels were assigned to textual fragments and were organized into categories. Saturation occurred after coding 40 semistructured interviews (eg, no new categories emerged). Existing and newly developed categories were merged into common themes in a consensus meeting (LB and CS). Disagreements were solved through discussion, together with author MV. To determine goal attainment, raw GAS scores were transformed into an individual mean GAS score (T-score). The T-scores included the attainment level and a potential weight assigned to the goals. T-scores of ≥50 indicate effective goal achievement [26].

## Results

### First-Order Process Data: Sampling Quality

#### Recruitment and Randomization

**Figure 1:**
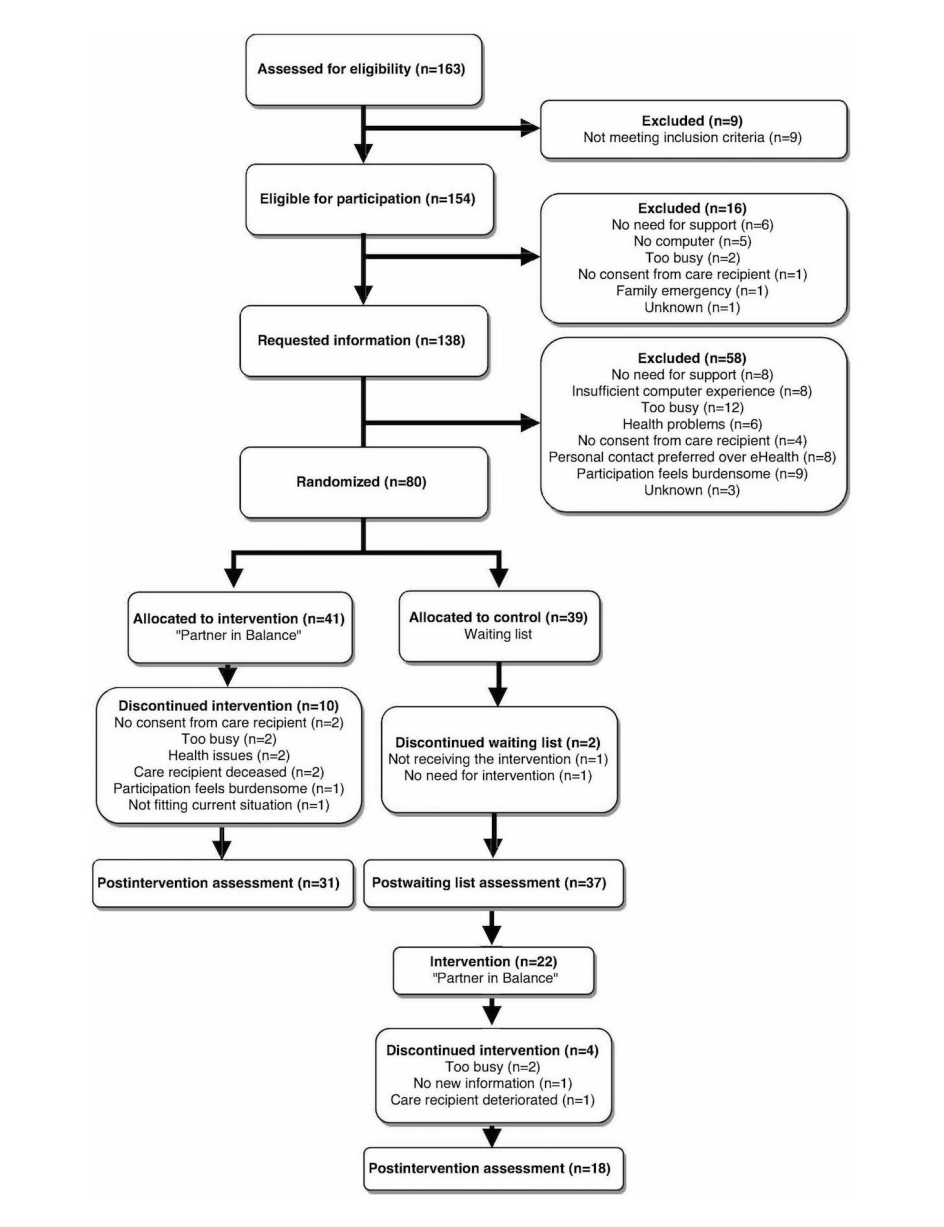
Study flowchart.

Caregivers were recruited from memory clinics (MUMC+, Elkerliek Hospital Helmond, Catharina Hospital Eindhoven), ambulatory mental health clinics (Virenze-RIAGG Maastricht, MET ggz Roermond), caregiver support services in the southern regions of the Netherlands, and the Dutch Alzheimer Association. A total of 163 caregivers were invited to participate. See Figure 1 for the study flowchart. If they expressed interest, family caregivers (n=138) received a detailed information letter. Of the information recipients, IC was signed by 58.0% (80/138). Of the 163 recruited caregivers, 154 were eligible for participation. The participation rate of eligible caregivers was 51.9% (80/154). Following the baseline assessment, the participants were randomly allocated to either PiB or the waiting-list control group. The researcher (LMMB) not involved in the assessments performed the allocation. A research assistant blind to the allocation conducted the assessments and recorded the blinding success and reason for the possible unmasking on the case record form. At T1, 68 participants had completed the postintervention or postwaiting list assessment and blinding was intact for 46% (31/68), unsuccessful for 49% (33/68), and for 7% (5/68), a conjecture of allocation was expressed.

#### Barriers and Facilitators for Recruitment

The interviewed coaches (n=10) reported that their caseload comprised several people with dementia living alone without a registered primary caregiver. This was listed as a primary recruitment barrier next to “caregiver does not own a computer” and “caseload only comprises caregivers of people with moderate to severe dementia.” Other barriers included concerns of burden for caregivers, high staff workload, and involvement in other caregiver support approaches. The coaches reported that younger caregiver age and being employed were facilitators for program recruitment because eHealth is best suited to those with a busy schedule and work-related computer literacy.

#### Reach

The caregivers were invited to participate by the clinician who treated their family member (n=122), were informed about the program’s existence by the Dutch Alzheimer Association n=26), or knew caregivers or family members already involved in the program (n=4). Others (n=11) requested information based on editorials in health magazines, local newspapers, and information stands in the southern parts of the Netherlands. The Dutch Alzheimer Association disseminated information about the program via the following: (1) monthly meeting spots for people with dementia and their caregivers, (2) newsletters, and (3) their website and social media platforms, including Facebook and Twitter.

### First-Order Process Data: Intervention Quality

#### Intervention Relevance and Feasibility: Coach Perspective

All coaches (n=13) completed the questionnaire and rated “PiB” as adequately feasible in daily practice (mean 4.5 on a scale of 1 to 5, standard deviation [SD] 0.5) and as fairly easy to integrate into their work-related activities (mean 4.4 [SD 0.5]). The program was rated as a useful addition for family caregivers (mean 4.5 [SD 0.5]) and for the coach as a professional caregiver (mean 4.2 [SD 0.8]). The mean time spent per participant was 6.2 hours (SD 1.5) spread over 8 weeks: intake session, 1.9 hours (SD 0.5); online feedback, 2.5 hours (SD 1.0); and evaluation session, 1.4 hours (SD 0.7). Qualitative analysis of the open-ended questions and the focus group interviews resulted into two themes: advantages for coaches and caregivers, and experienced difficulties and suggestions for improvement. Themes are described below and illustrated with quotations. Each quotation is assigned a code indicating the coach number in the trial.

#### Advantages for Coaches and Caregivers

The participants’ detailed input on the assignments enabled coaches to empathize with their situation and focus on their feedback. They reported a more profound relationship with the participant after the intervention. The flexibility to provide feedback whenever and wherever via email was considered positive; it fitted their busy schedule and offered them the time to reflect on their words. Coaches considered the face-to-face intake session crucial for developing a personal connection with the participant. Furthermore, they stressed that the examples offered recognition for participants, and the assignments increased awareness about behaviors and feelings. The program’s focus on quantifiable goals and possible solutions instead of problems was considered valuable for the participants. The availability of the information, assignments, and feedback after the intervention was seen as an advantage over mere face-to-face support. One coach stated:

By focusing on a particular problem or situation, other issues are raised that normally would not be addressed. It really interrupts the normal routine of both the client and coach. I noticed that it deepens the relationship you have with people.

C1

#### Experienced Difficulties and Suggestions for Improvement

Some coaches struggled with their level of direction in the self-management approach because they were accustomed to a proactive role and felt the urge to assist more than instructed for this PiB program. Goal setting during the intake session was considered difficult. However, coaches stressed the importance of goal setting and quantifying possible outcomes of program participation to make them tangible.

Several suggestions for improvement were mentioned. An increase in the user-friendliness of the instant messaging was suggested. Coaches preferred to provide feedback directly in the assignments over providing feedback in a separate message. Furthermore, a possible alarm function for crisis situations and follow-up care options were desired. To avoid confusion in the module structure, making modules available to caregivers one by one was suggested.

#### Intervention Relevance and Feasibility: Participant Perspective

The participants who completed the intervention (n=49) most often selected the available themes *communication with family member and environment* (44×) and *self-understanding* (44×), followed by *the changing family member* (39×), *acceptance* (36×), *coping with stress* (30×), *balance in activities* (24×), *insecurities and rumination* (21×), *focusing on the positive* (20×), and *social relations and support* (20×). Categories that emerged from the semistructured interviews were divided into the following four themes: program content, program structure, role of the personal coach, and target audience. Each quotation is assigned a code indicating the participant number in the trial.

#### Program Content

Most participants were satisfied with the content because it suited their current concerns. Specifically, the personalized assignments and challenges were appreciated. It was often mentioned that the content was not innovative but rather a confirmation of the participants’ conjectures. Some participants struggled with the primary focus on the caregiver because they felt the care recipient should change. One participant stated:

I think it is odd that the caregiver has to make changes, and my wife [person with dementia] does not have to learn or change anything.

P25

The participants reported increased awareness of their own behavior and its impact on the care recipient. The participants felt that they were challenged to explore their problems, uncover the causes, and actively seek solutions within the possibilities of the current situation. One participant stated:

You are forced to analyze your problems, but you do not linger in them because you have to look for possible solutions. Do not reach for the stars, but try realistic things. That helped me to put my situation and feelings into perspective.

P28

Some participants, however, were frustrated that not every situation has a solution, whereas others experienced a more accepting attitude. Furthermore, the participants appreciated and related to the examples from other caregivers who described their daily encounters with their loved one.

Suggestions for content improvement were also made. The inclusion of links to more disease-specific information was desired. Adding subtitles to the movie clips to watch them on mute was preferred when the care recipient was not aware of the caregiver’s participation.

#### Program Structure

The program structure of blending face-to-face contacts with online modules was experienced positively. The participants mentioned that the face-to-face contact personalized the program and increased their openness during the online assignments. One participant stated the following:

You need to see who is going to ask you these intimate questions. In my opinion that is a prerequisite for sending such personal information.

P68

It was stressed that the module structure facilitated the personal assignments, the examples fostered reflection, and the tips were used as input. Others reported difficulties fitting their answers into the assignment structure. Some participants mentioned using the website when they had time and often revisited the examples, their own answers, or the feedback. A printed workbook and an autosave option were considered desirable additions.

#### Role of the Personal Coach

The feedback was considered both supportive and critical at times, allowing participants to reflect on their answers. The participants emphasized that the coach was essential for motivation and questions. Knowing that someone was available to guide allowed them to feel less alone. One participant stated:

She [coach] made me think about things. You do not expect the feedback to be like the philosopher’s stone, but it is nice to get some confirmation and sometimes a critical note; “You are saying this, but how are you going to live up to it?” You are not doing it alone.

P03

Participants with a familiar coach reported an intensified relationship after working through the intervention together. Being able to speak freely online and becoming acquainted with the coach on a different level deepened their existing bond. Finally, the participants mentioned that coaches should not participate on the discussion forum because this was a safe zone for caregivers’ opinions.

#### Target Audience

Younger participants felt that the program should be specified to different subgroups. They could not identify with the older population in the examples because they were still employed and dealt with other issues in daily life, such as (young) children living at home. One participant stated the following:

As a younger and employed person I cannot identify with the older people and their struggles in the movie clips. It makes me feel alone.

P67

The discussion forum was not used because caregivers struggled with shame in the early stages, and sharing their story felt like a betrayal to the care recipient. Reading about other people’s misery was considered undesirable. Some participants mentioned that the course came too late for them. It was stressed that the program should be made available for all caregivers following a diagnostic disclosure. One participant stated:

I was exceptionally alert and active in my search for information, but it should be accessible for everyone. Do not wait until people ask for it. I considered it an “integration course” for caregivers.

P54

**Table 2:** Number of set goals per domain.

Number of set goals per domain (N=42)	Example of set goal per domain
Communication with care recipient (21)	“I want to stay connected to my spouse.”
Positive activities with care recipient (5)	“I want to find a TV program that is easy to follow so we can enjoy our evening together.”
Social support and contacts (10)	“I want to maintain our social connections”
Time alone and feeling guilty (16)	“ I want to go hiking twice a week for 1 hour by myself.”
Tension and anxiety (13)	“I want to be able to talk to my friends about my feelings as a caregiver.”
Role and relationship changes (9)	“I want to accept that it is normal to have an argument about the changes that we are facing.”
Feeling in control by gaining knowledge (8)	“I want to understand the behavioral changes and learn how to adapt to these changes.”
Positive thoughts and rumination (11)	“I want to worry less about the future and enjoy the positive moments we experience together.”’

#### Goal Attainment

Of the program completers in both groups (n=49), 42 participants set 93 goals in total. Five program completers were not able to set goals because they felt their care recipient should change and 2 completers missed their previously set goals because of coach turnover. Most participants (n=35) achieved ≥1 goals. Overall, 70 goals were attained (43 attained, 17 higher than expected, and 10 much higher than expected), and 22 goals were not attained (17 lower than expected and 5 much lower than expected). The mean T-score at baseline (set at −2 level) was 25.2 (SD 3.4, range 21.0-30.0). The mean achieved T-score at postintervention was 50.1 (SD 10.08, range 30.0-77.4). The mean set goals per participant were 2.2 (SD 1.1, range 1-4). To create an overview of the goals set by participants, they were categorized in domains. Table 2 shows the number of goals for each domain, with most goals set on communication with the care recipient, followed by planning time alone without feeling guilty about it, decreasing or preventing tension and anxiety, and obtaining social support.

#### Performance According to the Protocol

Intervention performance according to protocol comprised a face-to-face intake session, online modules over 8 weeks, individualized feedback via email for each module, and a face-to-face evaluation session. A total of 10 out of 13 coaches reported performance according to the protocol (77%), and 3 out of 13 reported deviations in intervention time, structure, and feedback (23%). Intervention time was reported to be longer (n=2) or shorter (n=1) than 8 weeks, and the module structure was consumed differently than intended (n=2) or feedback was given by telephone (n=2) or in person (n=1). Reasons to deviate from the protocol included caregiver pace and understanding of the program structure (n=3), illness (n=1), holiday leave (n=1), changes in work load and hours (n=1), personal family emergencies (n=1), and struggling to verbalize feedback in an email (n=1).

Regarding the dose delivered, out of the program completers (n=49), 87.8% (43/49) completed all 4 modules, 6.1% (3/49) completed 3 modules, and 6.1% (3/49) completed 2 modules. The tracked usage data showed 21,946 clicks per module, including exploring the website (2444 clicks), viewing the psychological educative information (3922 clicks), completing the assignments and change plan (8748 clicks), contacting the personal coach (6489 clicks), and visiting the discussion forum (310 clicks). The total intervention time ranged from 4 to 32 weeks (mean 13.9 [SD 6.8]). Reasons for intervention period variability were holidays, illness, busy schedules, and technical difficulties. Following the intervention period, 77.6% of the program completers (38/49) requested access to the additional modules with (16/49) or without (33/49) the coach at their disposal for questions.

### Second-Order Process Data: Implementation Knowledge

#### Implementation Components

The coaches (n=13) had a professional background as psychologist (n=7) or psychiatric nurse (n=6). During the regular supervision meetings, coaches shared experiences and asked for feedback. Support concerning the website and feedback content was requested outside the supervision meetings via email (n=13) and telephone (n=6) during the trial.

#### Barriers and Facilitators for Implementation

The directors of 22 dementia care organizations in the southeastern part of the Netherlands were asked to participate in the trial. The response rate of the organizations was 73% (16/22). Out of the responders, 63% (10/16) expressed interest in participating in the program. Refusal was based on upcoming reorganization (1/6), the lack of suitable caregivers (4/6), or the high workload of staff members (1/6). Out of the interested organizations, 40% (4/10) organizations choose to implement “PiB” and train staff members (psychologists or psychiatric nurses) to act as personal coaches. Furthermore, 6 organizations (60%) chose to refer caregivers to the coordinating center because of the high staff workload. Categories that emerged regarding implementation barriers and facilitators from the coach questionnaire and focus groups were divided into the following three themes: organizational aspects and financing, time and practical aspects, and the organization’s target population. The themes are described below and are illustrated with quotations, followed by a code indicating the coach number in the trial.

#### Organizational Aspects and Financing

Coaches mentioned that elderly care organizations in the Netherlands often file caregiver support under patient care, which could create problems for the implementation of caregiver support programs when the person with dementia is not registered. Additionally, financial cutbacks hampered professionals from adopting new support tools. The facilitating aspects included registration of the caregiver independent from the person with dementia, insurance compensation, and integration of online support in already provided caregiver support. One coach stated the following:

If we cannot register caregiver support, we cannot make time for it. Managers need to be convinced that caregiver support also benefits the person with dementia.

C3

#### Time and Practical Aspects

It was mentioned that unfamiliarity with the program could create a barrier for future implementation. Training and self-study were considered substantial personal time investments. The coaches suggested training all staff members as coaches and designating the program as regular care to facilitate implementation. One coach stated:

The program could easily be implemented as regular care if all staff members or a constant group of staff members were trained as coaches.

C4

#### Organization’s Target Population

Professionals mentioned that many health care organizations only treat people with moderate to severe dementia with severe comorbidity, or they expect the family caregivers not to be computer literate because of their advanced age, which hampers implementation of this type of caregiver support.

## Discussion

### Overview

This study described the process of PiB to explore its credibility and generalizability. First-order and second-order process data were evaluated from the perspective of both family caregivers (participants) and professionals delivering the intervention (coaches) to increase the understanding of the mode of delivery [30].

### Sampling and Randomization

The data on sampling quality showed that the participation rate of caregivers was 51.9%. Considering that the average response rate is 27% for caregiver research, our participation rate can be considered substantial [31]. However, these 51.9% only included eligible caregivers who were already familiar with the care parties involved in recruitment. Therefore, they may have been highly motivated and open to support [32]. Recruitment barriers were lack of computers, lack of need for intervention or additional support, etc, which were also reasons for the respondents to decline participation. During the early stages, caregivers may struggle with a fear of stigma and low acceptance [19]. This might explain why some participants struggled with accepting their own crucial role in any desired changes and why participants were not ready to openly discuss their issues in the discussion forum. However, the low use of the discussion forum could also be a consequence of the abundance of currently available online communication tools, eliminating the need for yet another form of online communication.

Young age and employment were considered recruitment facilitators. This finding is congruent with the findings of previous research stating that lower age correlated to higher eHealth literacy, that is, the skills and knowledge necessary to use online health resources [33,34]. However, seniors’ use of the Internet is expected to rise in the near future, increasing the accessibility of eHealth programs, such as PiB [35]. Furthermore, the results show that spouses and children of people with young onset dementia had difficulty identifying with the older caregivers in the examples. Previous research confirmed that younger caregivers struggle with different aspects in daily life compared with older caregivers [36]. It is essential to match program content to the specific needs of the target audience to maximize the benefit of a supportive intervention [6,37,38]. The thematic structure of the program allows for an add-on of specific themes for subgroups, such as caregivers of people with young-onset dementia.

At the postintervention assessment (n=68), the blinding of allocation was intact for only 46% (31/68) of the participants, which can potentially bias the estimation of effectiveness. However, blinding in psychosocial research can be challenging and it is rarely reported if blinding is maintained [39]. This item from the CONSORT may have been developed with studies of pharmacological treatments in mind, but blinding or masking may be unfeasible for certain aspects of psychosocial treatment studies [40]. Other important aspects of psychosocial RCTs need to be considered and reported but are not addressed in the CONSORT statement, for example, adequate reporting of treatment integrity (whether therapists were consistent in providing the specified intervention). Verification of treatment integrity, or fidelity, in outcome studies is essential to ensure that valid comparisons of replicable treatments can be made [41].

### Intervention Quality

The professionals were satisfied with the intervention being manageable considering their busy schedules, giving them time to focus on their feedback. They reported a more profound relationship with the caregiver; the program allowed them to empathize with the caregiver. However, the professionals considered the nondirective attitude toward the participants a challenge in the self-management approach. This issue deserves further exploration because the performance of the self-management health care provider is essential for the participant’s performance of self-management tasks and overall intervention effectiveness [42]. It was previously argued that health care providers do not always support self-management education because this is not part of their attitudes, beliefs, or regular care practice [27]. To maximize the effects of self-management programs, increasing the essential clinical competences of health care providers to provide tailored self-management support [43] and raising awareness of the benefits of evidence-based self-management programs for their target population [27] are recommended. For example, this study showed that both participants and coaches mentioned a more profound relationship with one another, which was also demonstrated in a previous blended-care intervention for depression [44]. Previous research confirmed that the opportunity to reflect on one’s feelings anonymously in a private and safe environment is easier than doing so in person, but face-to-face contact increased caregiver openness and coach empathy [7]. The participant compliance with all 4 modules was high (87.8%), which could be explained by the motivational aspect of having a coach [7]. The varying intervention period and dose may influence the effectiveness of the intervention [41]. However, reasons for protocol deviations were diverse and not uncommon for informal caregivers and elderly participants (eg, caregiver pace, time constraints, and illness) [31]. In self-management interventions, the participant is in control and should therefore be allowed to complete the modules at his own pace [27,45]. Furthermore, complex interventions such as PiB are often designed to be implemented with some flexibility to accommodate differences among participants [46].

### Implementation

PiB was evaluated within multiple organizations with coaches from different backgrounds who received training and supervision in self-management and blended care. A relatively high response rate of organizations was found (73%), which could be attributed to the current demand of health care insurance for care organizations to provide eHealth [47]. However, a lack of financing and time could hamper the implementation. To overcome the experienced barriers and implement the program on a larger scale, awareness of the benefits of blended care self-management programs is required in addition to the training of self-management skills for the health care providers. The MRC Framework suggests that implementation should be considered during the first phases of intervention development and evaluation [48]. Involving stakeholders in technological development and evaluation can facilitate implementation in different care settings [49]. As complex interventions are influenced by context, several psychosocial interventions show different results in different settings or countries [40]. Additional information about the process of the implementation is crucial to understanding why an intervention is effective in one setting and not in another [46]. Before implementing the program on a larger (international) scale, barriers and facilitators for implementation should be identified with regard to possible differences based on organizational and cultural contexts. PiB was evaluated within different settings with coaches of different backgrounds delivering the intervention. To evaluate whether the background of the coach has any influence on the intervention outcome, this variable can be considered in the effect analysis. Additionally, the higher rate of dropout in the intervention group showed that this program can be considered burdensome. eHealth interventions in general are not appropriate for caregivers who are not computer literate or have more practical care needs. Several other factors influence the interaction between people with dementia and their caregivers, for example, caregivers’ personalities, psychological well-being, and the psychological symptoms of the care recipient. For instance, our results showed that this early-stage intervention came too late to help some caregivers. Caregivers were included based on the stage of the disease of their loved one, but some caregivers had been struggling with insecurities for years while the stage of the disease was still considered “early.” This highlights the need for tailored interventions, not only for the stage of the disease but also for the personal experiences, capacities, and other factors that may contribute to the intervention efficacy. Future research should consider including larger samples to examine the impact of eHealth interventions for subgroups of caregivers to tailor the care offered more efficiently [5]. However, in this study, a relatively high response and participation rate was found, indicating that having the option to choose this type of caregiver support is needed at a minimum. An active role of health care professionals in outlining care and support options early in the dementia process is recommended. Furthermore, sustainability of long-term intervention effects should be evaluated, and a cost-consequence or cost-effectiveness analysis should be conducted to inform decision makers of the value of PiB.

### Methodological Considerations

Several study limitations need to be considered. First, in our study protocol, deviations were measured with the self-report questionnaire for coaches. Previous research measuring protocol deviations with self-report questionnaires and ratings based on audio recordings found large discrepancies between the two measurements, indicating that professionals may not always be aware of their level of treatment fidelity [50]. Second, tracked usage data were measured in clicks. Clicks represent page views, but this clickstream method has a large disadvantage; several people who click on a page do not necessarily read it. Furthermore, 1 in 3 visitors spend less than 15 seconds reading the page, so a measured click does not automatically mean that the attention of the visitor is directed to the information on the page that is being viewed [19]. However, the results showed that the participants spent most of their time on the assignments and change plans and email contact with their coach, which represent the essential elements of a blended-care program [7,44]. Third, GAS was used to rate goal attainment. Goal setting and rating are considered a therapeutic task and were therefore performed by the coaches during the face-to-face sessions. Future research could consider setting and rating goals by an independent research assistant in all treatment arms to consider GAS as an outcome measure. However, GAS is a challenging evaluation method when not all participants set or evaluate goals, goals change during the process, or participants lack insight, communication skills, or the capacity to specify goals, and therefore should be used merely as a complementary scale [51].

### Conclusions

The participants and professionals were satisfied with the intervention, but adapting the content to specific subgroups such as younger caregivers was recommended. Implementation of the program requires more awareness of the benefits of blended care self-management programs and training in tailored self-management skills for the health care provider. Overall, PiB can be considered a valuable addition to the existing caregiver support because it is tailored to the needs of the target audience and deepened the coach-caregiver relationship.

## Appendix

Multimedia Appendix 1 Structured registration form.

Multimedia Appendix 2 Coach questionnaire.

## Abbreviations

eHealth: electronic health
GAS: goal attainment scaling
IC: informed consent
PiB: Partner in Balance
MRC: Medical Research Council
MUMC+: Maastricht University Medical Center
RCT: randomized controlled trial
SD: standard deviation
