# Supplementary material for: Implementation of the Blended Care Self-Management Program for Caregivers of People With Early-Stage Dementia (Partner in Balance): Process Evaluation of a Randomized Controlled Trial
Source: J Med Internet Res. 2017 Dec 19;19(12):e423. doi: 10.2196/jmir.7666 (PMC5750419; doi:10.2196/jmir.7666)
Supplement: Multimedia Appendix 1 [file jmir_v19i12e423_app1.pdf]

Multimedia appendix 1. Structured registration form coaches Partner in Balance

Participant number: ________

Action undertaken	Date	Time spent	(If applicable) Who took initiative?	Topics discussed	Other remarks
Preparation<br>- Attending coach training<br>- Reading protocol<br>- Attending coach intervision meetings					
Intake session<br>- Getting acquainted<br>- Needs assessment<br>- Setting GAS goals<br>- Choosing modules + adding modules to account<br>- Explaining online procedure<br>- Contact moment agreements<br>- Providing login information					
Online period<br>- Responding to online assignments (email)<br>- Other online help (support requests, reminders)<br>- Telephone contacts (if applicable)					
Evaluation session<br>- General evaluation of modules and experience with the program<br>- Evaluating GAS goals<br>- Identify need for additional modules or types of aid					
