# Supplementary material for: Implementation of the Blended Care Self-Management Program for Caregivers of People With Early-Stage Dementia (Partner in Balance): Process Evaluation of a Randomized Controlled Trial
Source: J Med Internet Res. 2017 Dec 19;19(12):e423. doi: 10.2196/jmir.7666 (PMC5750419; doi:10.2196/jmir.7666)
Supplement: Multimedia Appendix 2 [file jmir_v19i12e423_app2.pdf]

Multimedia appendix 2: Coach questionnaire

	Completely disagree	Disagree	Neutral	Agree	Completely agree
1. The program is feasible for coaches	1	2	3	4	5
2. I can combine the coach responsibilities with my usual activities	1	2	3	4	5
3. The program is a valuable contribution for caregivers	1	2	3	4	5
4. The program is a valuable contribution for coaches/professionals	1	2	3	4	5

5. Was the program delivered according to protocol (in +/- 8 weeks, feedback after each module, face-to-face intake and evaluation session). If not, what was performed inconsistent with the protocol and why?

6. How much time, on average, did you spent coaching one participant? (Based on the structured registration form)

7. What are the advantages of the program?

8. What are the disadvantages of the program? Points for improvement?

Implementation

9. What are the barriers for implementation of the program in your organization?

10. What could be possible facilitators for future implementation of the program within your organization?
